# The epigenetic regulation of *HsMar1*, a human DNA transposon

**DOI:** 10.1186/s12863-019-0719-y

**Published:** 2019-02-14

**Authors:** Sylvaine Renault, Murielle Genty, Alison Gabori, Catherine Boisneau, Charles Esnault, Thomas Dugé de Bernonville, Corinne Augé-Gouillou

**Affiliations:** 1 EA 6306 Instabilité génétique et cancer, Université de Tours, UFR Sciences et Techniques, UFR Pharmacie, 31 Avenue Monge, 37200 Tours, France; 2 UMR 1253, iBrain, University of Tours, INSERM, Tours, France; 3 UMR CITERES CNRS 7324, Université de Tours, 35 Allée Ferdinand de Lesseps, 37200 Tours, France; 4 EA 2106 BBV, UFR Sciences et Techniques, UFR Pharmacie, 31 Avenue Monge, 37200 Tours, France

**Keywords:** Mobile DNA, Epigenetic, Network, Transposon

## Abstract

**Background:**

Both classes of transposable elements (DNA and RNA) are tightly regulated at the transcriptional level leading to the inactivation of transposition via epigenetic mechanisms. Due to the high copies number of these elements, the hypothesis has emerged that their regulation can coordinate a regulatory network of genes. Herein, we investigated whether transposition regulation of *HsMar1*, a human DNA transposon, differs in presence or absence of endogenous *HsMar1* copies. In the case where *HsMar1* transposition is regulated, the number of repetitive DNA sequences issued by *HsMar1* and distributed in the human genome makes *HsMar1* a good candidate to regulate neighboring gene expression by epigenetic mechanisms.

**Results:**

A recombinant active *HsMar1* copy was inserted in HeLa (human) and CHO (hamster) cells and its genomic excision monitored. We show that *HsMar1* excision is blocked in HeLa cells, whereas CHO cells are competent to promote *HsMar1* excision. We demonstrate that de novo *HsMar1* insertions in HeLa cells (human) undergo rapid silencing by cytosine methylation and apposition of H3K9me3 marks, whereas de novo *HsMar1* insertions in CHO cells (hamster) are not repressed and enriched in H3K4me3 modifications. The overall analysis of *HsMar1* endogenous copies in HeLa cells indicates that neither full-length endogenous inactive copies nor their Inverted Terminal Repeats seem to be specifically silenced, and are, in contrast, devoid of epigenetic marks. Finally, the *setmar* gene, derived from *HsMar1*, presents H3K4me3 modifications as expected for a human housekeeping gene.

**Conclusions:**

Our work highlights that de novo and old *HsMar1* are not similarly regulated by epigenetic mechanisms. Old *HsMar1* are generally detected as lacking epigenetic marks, irrespective their localisation relative to the genes. Considering the putative existence of a network associating *HsMar1* old copies and SETMAR, two non-mutually exclusive hypotheses are proposed: active and inactive *HsMar1* copies are not similarly regulated or/and regulations concern only few loci (and few genes) that cannot be detected at the whole genome level.

**Electronic supplementary material:**

The online version of this article (10.1186/s12863-019-0719-y) contains supplementary material, which is available to authorized users.

## Background

Transposable elements (TEs) are mobile genetic elements representing a prevalent part of eukaryotic genomes, including human. They are known to display significant genetic consequences, promoting various types of mutation such as disrupting genes (upon neo-insertions) or inducing recombination between homologous sequences at divergent loci. Beyond these foreseeable consequences in view of TEs mobility and/or amplification, other more unexpected effects relying on TEs occurrence were illustrated during the two last decades. First, TEs possess their own regulatory sequences, and then could alter the normal expression pattern of neighboring genes. It has also been shown that amplification of various TEs family can provide new gene regulatory networks [1]. Finally, exaptation of several TEs is believed to drive various genetic innovations [2].

The genetic consequences of TEs moving around genomes via an RNA intermediate (also called retrotransposons) were widely illustrated in human since this class of TEs represents 97% of human TEs and because some are still active and responsible for several genetic diseases. 0.3% of human TE insertions have been suggested for causing a disease, i.e. one insertion in every 20–100 live births, and approximately 96 new transposition events are directly linked to single-gene diseases [3, 4]. Because genomes accommodate millions of TEs that putatively disrupt “normal” functioning, TE activity (generally speaking) needs to be controlled, a role that is mainly undertaken by epigenetic mechanisms [5]. These mechanisms (from imprinting and X inactivation, to position effect variegation) are correlated to retro-transposons and associate to well-known epigenetic pathways (RNAi, DNA methylation, and specific histone modifications) [6, 7] repressing retro-transposons activity in normal human cells. In contrast, DNA methylation can be abolished in cancer cells, opening the possibility for retro-transposons to be activated and to affect the integrity of the cell (reviewed in [8]). Even if few studies investigated histone modifications related to human retro-transposons, a global loss of H4K16me and H4K20me3 has been associated to repetitive elements [9, 10], and the spread of these modifications to adjacent regions has been observed in plants, fungi, and mouse [5].

Less is known about the genetic consequences of TEs moving around genomes via a DNA intermediate (also called transposons) that represent 3% of human TEs. Studies in other mammalian species suggest that transposons are also controlled by epigenetic mechanisms, potentially differently than retro-transposons. For instance, *Sleeping Beauty* (SB) transposition is easier when the transposon is methylated [11, 12] and this is also the case when SB excision occurs from genomic loci. More surprisingly, heterochromatin formation seems to facilitate SB excision when the needed enzyme (the transposase) is supplied in *trans* [13]. This counterintuitive observation may rely on the DNA/protein complex assembly needed for SB transposition, for which chromatin conformation is assumed to be determinant. In this model, old insertions (located in heterochromatin regions) are mainly silenced by repressing the transposase gene expression. Transposition could be reactivated upon induced chromatin changes, after genomic stress for instance.

Several groups seek to understand how TEs silencing takes place upon genome invasions and exaptation of elements. This requires developing new biological models to mimics TE invasion in a naive context, to address de novo insertions. For the human L1 retro-element, this was done by studying pseudo-founder transgenic mice and their progeny [14]. De novo L1 integrations undergo rapid silencing by dense cytosine methylation in pluripotent mouse embryonic stem (ES) cells, and silencing is retained in several somatic tissues of adult founder mice. Interestingly, L1 copies that are mobilized later in somatic development and differentiation (like in cancer cell lines) are reversibly silenced by histone deacetylation, suggesting that the cellular contexts of L1 retro-transposition can determine expression or silencing of newly integrated sequences. By contrast PiggyBac (PB), a DNA transposon, reveals relatively stable and robust expression without apparent silencing in ES cells [14].

Herein, we describe how human DNA transposons are regulated in two contexts: endogenous existing copies and de novo insertions in a naive background. DNA transposons were highly active during mammalian radiation and early primate evolution, with no evidence of elements younger than approximately 37 My [15]. Among them, *mariner* elements were amplified 45 My ago and the *HsMar1* sub-family is probably the only one to display a current quite active copy. The “modern” human genome contains about 250 defective *HsMar1* copies (almost full-length), beside a domesticated copy, which codes (together with a histone-methylase gene) a chimeric protein called SETMAR [16]. The MAR domain of SETMAR displays quite all the properties of the *HsMar1* transposase, except for the ability to cleave the first DNA strand upon excision [17]. This difference prevents SETMAR from promoting *HsMar1* transposition around the human genome. Next to the full-length copies, the human genome also contains thousands of miniature *HsMar1* (from which hsa-mir-548 come from [18]) and solo TIRs (TIRs, for Terminal Inverted Repeats, are the target sequences for the transposase to provide mobility; they are 30 bp sequences usually located at the end of the full-length elements). The chromatin status of these *HsMar1* relics, if regulated, may impact thousands of loci, and their neighboring genes. Old endogenous *HsMar1* copies may therefore provide a putative regulatory network.

The availability of “reconstructed” *HsMar1* active copies provides opportunities to perform de novo insertions in either naive genomes (non-human) or *HsMar1*-containing genomes (human). Doing so, we demonstrate that de novo *HsMar1* insertions in HeLa cells (human) undergo rapid silencing by cytosine methylation and affixing of H3K9me3 marks, whereas de novo *HsMar1* insertions in CHO cells (hamster) are not repressed and enriched in H3K4me3 instead of H3K9me3 marks, and transcriptionally active. The overall analysis of *HsMar1* old endogenous copies indicates that neither full-length endogenous inactive copies nor TIRs seem to be specifically silenced; in contrast, they are devoid of epigenetic marks. Taking into account the putative existence of a network associating *HsMar1* old copies and SETMAR, two non-mutually exclusive hypotheses are proposed: active and inactive *HsMar1* copies are not similarly regulated or/and regulations concern only few loci (and few genes) and cannot be detected at the whole genome level.

## Results

### Genomic background conditions *HsMar1* activity

**Fig. 1:**
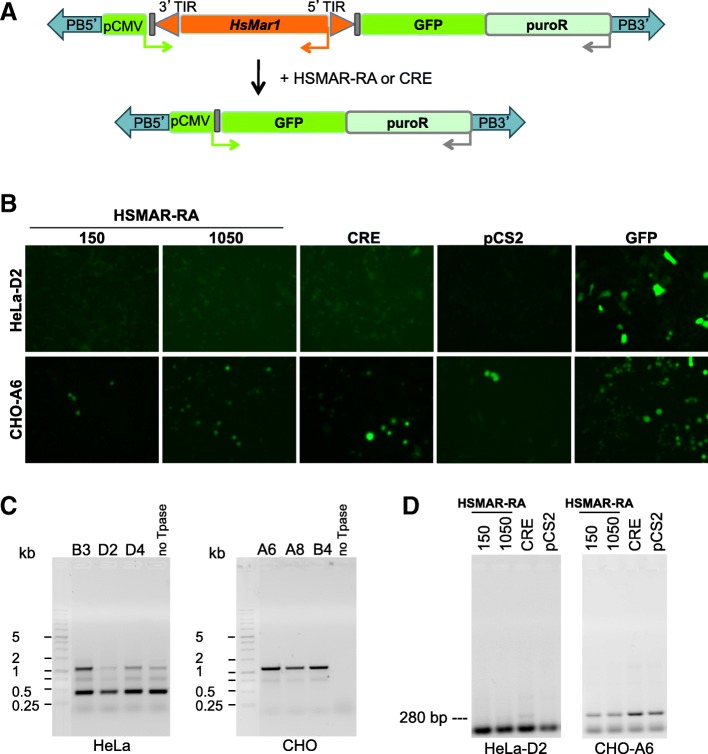
*HsMar1* excision in two genetic backgrounds: human (HeLa cells) and non-human (CHO cells). **a** *HsMar1* excision cassette before (top) and after (down) the expected *HsMar1* excision. PiggyBac (PB) TIRs are represented as blue arrows ending the cassette; the *HsMar1* recombinant copy is drawn in orange with its 5′ and 3’TIRs (orange arrows) and its transposase (Tpase) (orange rectangle); the LoxP recombination sites of CRE recombinase are in grey and GFP in green. The three promoters (pCMV, endogenous *HsMar1* and that driving the puromycin resistance gene) are shown as thin arrows coloured in green, orange and grey respectively. **b** Excision assays exemplified for two cell lines (HeLa-D2 and CHO-A6). HeLa or CHO were transfected with HSMAR-RA (150 or 1050 ng), CRE (150 ng) expressing plasmid or an empty plasmid (pCS2). Transfection efficiency is controlled by transfecting a GFP expressing plasmid. 48 h after transfection, excision is controlled by GFP expression. **c** RT-PCR analyses of recombinant cell lines (for HeLa: B3, D2 and D4, left panel and for CHO: A6, A8 and B4, right panel) and their respective empty cell lines (no Tpase). BET-stained agarose gels are shown. Bands detected in HeLa cells are a mixed of PCR products obtained from both endogenous and recombinant *HsMar1* copies. **d** Excision sites amplification in recombinant HeLa-D2 and CHO-A6 cell lines 48 h post- transfection; the different expressing plasmids use to promote excision are indicated above and amounts are as in (B) (pCS2: control plasmid). BET-stained agarose gels of PCR products are shown. The expected band is pinpointed (left margin)

To follow the transposition of a complete and active *HsMar1* element, a tool was designed to examine the first step of transposition, the excision. An excision cassette was composed of an active *HsMar1* copy inserted in opposite orientation between the CMV promoter (pCMV) and the GFP coding sequence, thus preventing the expression of GFP. LoxP sequences were added at both *HsMar1* ends. Upon *HsMar1* excision (provided in *trans* by HSMAR-RA, the reconstructed active *HsMar1* transposase or by CRE expression), pCMV allows the expression of GFP in recombinant cells (Fig. 1a). The assay was designed in order to block GFP expression in absence of *HsMar1* excision. GFP has his own start codon allowing its expression if *HsMar1* was removed between the pCMV and the ORF of GFP. The construct also contains a selection marker (the puromycin resistance gene), the whole being enframed by PiggyBac ends. The excision cassette was introduced in HeLa and CHO genomes using PiggyBac transposition. Clonal recombinant cell lines were established and the presence of the excision cassette was controlled by PCR and sequencing. Each recombinant line contained at least one copy of the excision cassette (Additional file 1: Data S1).

Shortly after having established individual cell lines (five passages), excision assays were performed using various amount of HSMAR-RA or CRE expressing plasmids. The empty pCS2 plasmid was used as control. Among the 22 established HeLa cell lines, none provided GFP expression when transfected with an HSMAR-RA expressing plasmid, and only eight provided low GFP expression when transfected with a CRE expressing plasmid. No GFP expression was detected in controls (Fig. 1b and Table 1). In contrast, all the eight established CHO cell lines display a high level of GFP expression when transfected with either HSMAR-RA or CRE expressing plasmids (Fig. 1b and Table 1) and low GFP expression in controls. The excision efficiency of individual cell lines was quantified (Table 2) confirming that HeLa cell lines are not competent for *HsMar1* excision, whereas CHO cell lines are.

**Table 1:** Number of independent HsMar1 transgenic cell lines (HeLa and CHO) showing excision according to different conditions of transfection

	Hsmar1 transgenic cells lines	Excision after transfection with			
		No DNA	pCS2	CRE	HSMAR-RA
HeLa	22	0	0	8	0
CHO	8	7	8	8	8

**Table 2:** Percentage of cells per cell line (HeLa or CHO) showing excision according to different conditions of transfection

	No DNA	pCS2	CRE	HSMAR-RA
HeLa	0	0	1%	0
CHO	0–22%	0.8–30%	8–100%	6–50%

The detection of GFP signals in the absence of recombinases (HSMAR-RA or CRE) provided in *trans* strongly supports the idea that the recombinant *HsMar1* cassette is expressed in CHO, allowing the transcription and the translation of the transposase encoded by the recombinant copy. This was confirmed by RT-PCR (x-primers in Additional file 1: Table S1) in the absence of transfected HSMAR-RA (Fig. 1c) in both HeLa and CHO recombinant cell lines (B3, D2, D4 and A6, A8, B4 respectively) or empty (e) cell lines (as controls). As expected, CHO cells used as control did not express *HsMar1* transposase mRNA (endogenous *HsMar1* are restricted to the anthropoid lineage). In contrast, HeLa cells used as control showed expression of *HsMar1* transposase mRNA from endogenous *HsMar1.* Human cells contain about 250 full-length *HsMar1* copies, all of them containing deletions or mutations preventing the production of an active transposase. The detection of *HsMar1* mRNA resulted from the activity of its endogenous promoter, which is sufficient to drive the transcription of a remnant mRNA [19]. This was confirmed by the detection of the full-size PCR product (1.1 kb) in recombinant CHO cell lines (A6, A8, B4 – Fig. 1c, right panel). Beyond the full-size expected mRNA, smaller PCR products are also detected in HeLa cells, originating from deleted transposons. This “endogenous background” may mask the detection of PCR products coming from the recombinant copy (Fig. 1c, left panel, B3, D2, D4 versus “no Tpase”).

Finally, excision landmarks were validated by PCR analysis using primers anchored in pCMV and GFP regions (e-primers in Additional file 1: Table S1). The expected 280-bp fragment was only faintly detected in HeLa cells transfected by the CRE expressing plasmid, but gave a significant signal in all conditions for CHO cell lines (Fig. 1d). Excision specific signatures were confirmed by sequencing (not shown). We have excluded that the lack of *HsMar1* excision was due to a lack of active transposase (HSMAR-RA) expression in HeLa cells. As expected, the active transposase is correctly expressed under our experimental conditions, in accordance with the amount of plasmid used for transfection (Additional file 1: Data S2).

The analysis of sequences surrounding the integration sites in HeLa cells did not show specific DNA elements as defined in ENCODE database, which could explain the absence of transposition in these cells. All together these first results strongly support that the genetic background of cells (naive or not for *HsMar1*) influences the capability of transposition of neo-inserted copies.

### *HsMar1* activity is controlled by epigenetic mechanisms

As mentioned before, retro-transposons are subjected to inactivation by various epigenetic pathways. Conversely, little is known about epigenetic regulations of DNA transposons in somatic cells. Our first results suggested that the main difference between the behavior of the *HsMar1* excision cassette in CHO and HeLa cells relied on the absence of endogenous *HsMar1* copies in CHO. We therefore hypothesize that epigenetic mechanisms control *HsMar1* transposition in HeLa cells, independently of the regulation of the transposase expression.

**Fig. 2:**
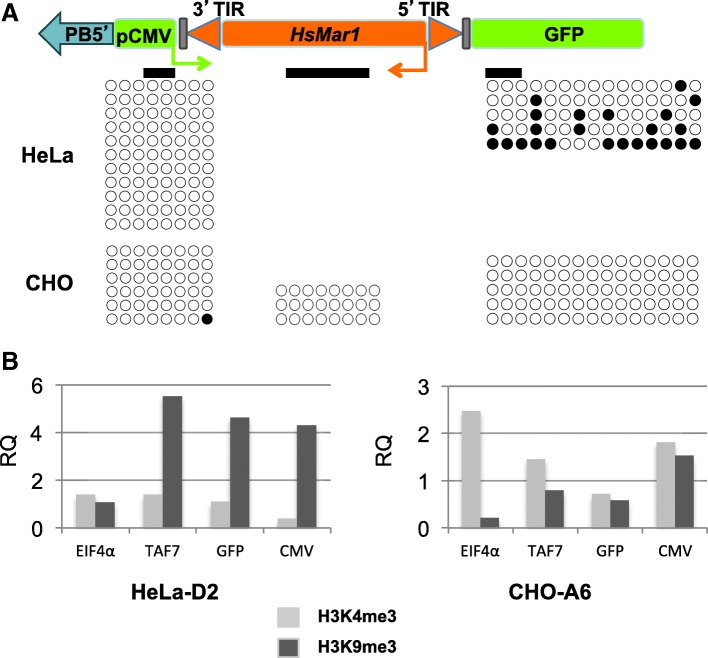
**a** Bisulfite conversion analysis of the *HsMar1* cassette present in recombinant HeLa and CHO cell lines. Bold black lines indicate the three analysed regions (pCMV, GFP and *HsMar1*). Three transgenic lines were analysed for methylation for each cells lines and the compilation of the results is presented: one round corresponds to a dinucleotide CpG present in the analysed fragment (black: mCpG; white: CpG). **b** Level of H3K4me3 or H3K9me3 relative to total histone H3 on different sequences: EIF4α, TAF7, GFP and pCMV present in excision cassette in two cell lines HeLa-D2 and CHO-A6. Histone marks present on GAPDH were used as references for comparison and expressed as relative mark deposition to GAPDH. H3K4me3 deposition is in light grey and H3K9me3 deposition in dark grey

We measured DNA methylation using conventional bisulfite conversion followed by PCR amplification and sequencing on three recombinant cell lines of each type (CHO and HeLa). Three regions within the *HsMar1* excision cassette were analysed: the CMV promoter, an internal *HsMar1* fragment and the flanking GFP sequence (Fig. 2a). In HeLa cells, we did not succeed to design primers that discriminate the endogenous *HsMar1* copies from *HsMar1* cassette and also could amplify several CpG. In fact, the endogenous *HsMar1* copies masked any alterations on the internal *HsMar1* fragment. In HeLa cells, the pCMV locus showed no DNA methylation whereas the GFP sequence was methylated. In contrast, CHO cells displayed no DNA methylation at whatever the part of the excision cassette. Using the ENCODE database we have verified that the CpG in the HeLa genome at the insertion loci were not methylated before the integration of the *HsMar1*. Since they were methylated after *HsMar1* integration, we assume that the detected mCpG were specifically induced by the cassette.

DNA methylation is often sustained by histone post-translational modifications, especially with deacetylation of histones H3 and H4, loss of H3K4me3, gain of H3K9me3 and H3K27me3 [9, 20, 21]. We have performed ChIP analyses on HeLa and CHO recombinant cell lines to detect the most frequent modifications, that is: H3K4me3 marks for activation and H3K9me3 marks for inactivation (Fig. 2b). As reference genes, *GAPDH* was used as an internal control, *TAF7* as associated with H3K9me3 and *EIF4α* as associated with H3K4me3, both in HeLa and CHO cells [22, 23]. HeLa cells displayed the expected results for both controls, whereas in CHO cells only *EIF4a* matched the expected data. In contrast, *TAF7* is not associated to H3K9me3. Since nothing is known about *TAF7* regulation in hamster, we assume that it is different from that observed in human cells without impairing our analysis. Our results clearly indicated that both GFP and pCMV sequences had obtained H3K9me3 marks in recombinant HeLa cells, contrary to recombinant CHO cells. Thus, the inactivation of the *HsMar1* cassette in HeLa cells is supported by both DNA methylation and H3K9me3 modification.

### Is the whole *HsMar1* network controlled by epigenetic mechanisms?

**Fig. 3:**
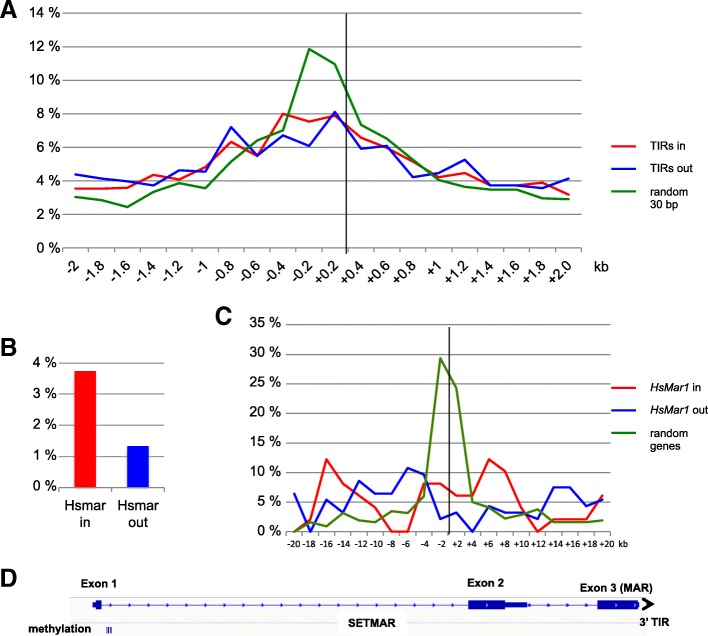
**a** Percentage of methylated MspI sites (mCpG) over all MspI sites within 0.2 kb intervals around TIRs present inside (TIRs in, red) or outside genes (TIRs out, blue) or random 30 bp sequences (green). **b** Percentage of *HsMar1* containing mCpG present inside (*HsMar1* in) or outside genes (*HsMar1* out). **c** Percentage of mCpG over all CpG within 0.2 kb intervals around *HsMar1* present inside (*HsMar1* in) or outside genes (*HsMar1* out) or random genes. **d** mCpG for *SETMAR.* The arrow represents the 3’ TIR present in *SETMAR*

The results presented so far only concerned the recombinant *HsMar1* copy added within genomes by recombination. We wanted to verify whether the old endogenous *HsMar1* copies or TIRs behave as the recombinant copy does. The human genome contains 231 “full-length” *HsMar1* elements, which vary in length between 1000 and 1300 bp. The ancestral reconstructed sequence is 1287 pb long, and the current genomic full-length copies are inactive since they accumulated various micro-mutations (insertions-deletions-substitutions). The 231 full-length elements display 80 to 95% identity with the ancestral sequence. Among them, 88 (about 39%) colocate within genes. We have also looked at *HsMar1* TIRs (HsTIRs) that are short inverted sequences of 30 bp ending the transposon. They are known to act as target sequences for the transposase during the transposition process and, for the 5’TIR, to be part of the *HsMar1* endogenous promoter [19]. The human genome contains about 12,500 HsTIRs, including those inside full-length *HsMar1* copies, those organized in miniature tandem repeats and solo TIRs. 3952 have conserved the ancestral length of 30 bp of which 39,9% colocate within genes. Other TIRs are shorter, but remain detectable up to 15 bp. If the endogenous copies are regulated as the recombinant copy does, the vicinity of genes evokes a potential role in regulating gene expression. Surprisingly, the 30 bp TIR sequences were well conserved by evolution for 45 My and even if only 6.2% are identical to the ancestral sequence, 67% of the changes relate to positions 24–25, which correspond to a CpG dinucleotide, suggesting that *HsMar1* TIRs have been subjected to CpG shortage. Since mCpG are frequently deaminated to TpG on both strand, the evolutionary effect will be a progressive loss of CpG dinucleotides, and a concomitant replacement by TpA dinucleotides, precisely what is observed on HsTIRs (not shown). Small HsTIRs may equally be affected by mCpG. From ENCODE database, we have analysed the HsTIRs CpG methylation environment in the previously published HeLa-S3 genome (Fig. 3a). RRBS data, which detect methylation on *MspI* restriction sites, were used. Since *HsMar1* TIRs do not contain an *MspI* restriction site, *MspI* methylation sites present in 200 bp intervals on both sides of TIRs were counted and expressed as a percentage of all *MspI* sites (Fig. 3a). The repartition of mCpG is significantly different around HsTIRs (whatever their positions towards genes) and around random 30-bp sequences (χ2 test, *p* > 0,0001): less mCpG are detected around HsTIRs than anywhere else in the genome.

Since the CpG near the recombinant *HsMar1* promoter were found methylated (Fig. 2a), we have looked for methylated CpG (mCpG) inside and near the 231 endogenous *HsMar1* full-length copies. The percentage of *HsMar1* presenting CpG methylation was calculated from RRBS data (Fig. 3b). Few *HsMar1* were methylated; among them, *HsMar1* inside genes (red line) appear more methylated than *HsMar1* outside genes (blue line) but these differences were not statistically significant (χ2 test, *p* > 0.05)(Fig. 3b). The methylation around 250 random-selected expressed genes (coding or not), from the GencodeV27 database, was similarly analyzed (Fig. 3c, green line). In this case, the distribution of mCpG appeared to focus within a 2 kb window corresponding to the promoter, taking in account that genes are not oriented in this analysis. Such distribution was not observed concerning *HsMar1* copies (Fig. 3c, red and blue lines), showing that *HsMar1* are not regulated by DNA methylation as are other expressed genes.

Finally, we examined the DNA methylation status of *SETMAR*, a fusion gene in which exon 3 is made of a full-length *HsMar1* element lacking the 5’ TIR (Fig. 3d). Importantly, *SETMAR* third exon encodes the only active *HsMar1* transposase of the human genome. The only restriction to exon 3 activity is its inability to cleave the DNA first strand, a step needed to excise the element from its donor site [17]. In the Hela-S3 genome, mCpG are found within the first exon and intron, near the promoter, as for other expressed genes (Fig. 3c). The 3′ end of the MAR domain (including the 3’ TIR) was found unmethylated, similarly to the 3′ end of the recombinant copy. Our findings indicated that “old” *HsMar1* TIRs and full-length copies were specifically less methylated than expressed genes. Interestingly, this did not seem to be the case for newly integrated copies, as mimicked by the recombinant ones.

**Fig. 4:**
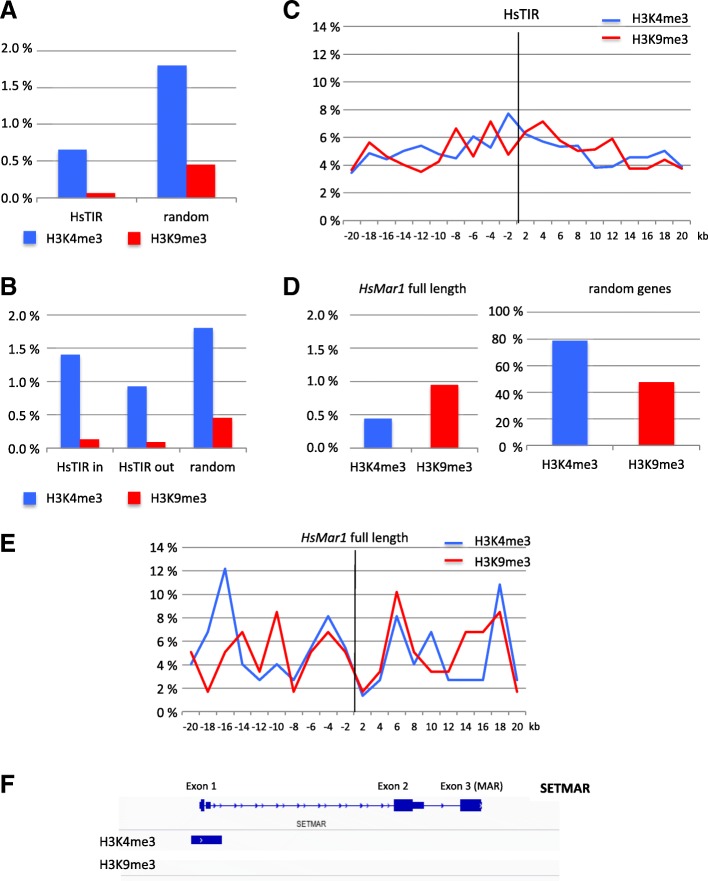
**a** Percentage of HsTIRs and random 30 bp sequences associated to H3K4me3 (blue) and H3K9me3 (red) in HeLa-S3 cells. **b** Percentage of 30 pb TIRs present inside or outside genes or random 30 bp sequences associated to H3K4me3 (blue) and H3K9me3 (red) in HeLa-S3 cells. **c** Percentage of H3K4me3 (blue) and H3K9me3 (red) marks in 2-kb intervals spanning a 40-kb window around HsTIR sequences (position “zero”) in HeLa-S3 cells. **d** Percentage of *HsMar1* (left panel) and random genes (right) associated to H3K4me3 (blue) and H3K9me3 (red) in HeLa-S3 cells. **e** Percentage of occurrence of H3K4me3 (blue) and H3K9me3 (red) marks in 2-kb intervals spanning a 40-kb window around *HsMar1* in HeLa-S3 cells. **f** H3K4me3 and H3K9me3 status of SETMAR. The blue rectangle corresponds to the position of H3K4me3 in *setmar* gene

By analysing ChIP results obtained from ENCODE in HeLa-S3 cells, we then addressed whether endogenous *HsMar1* copies and TIRs were associated to H3K9me3 and/or H3K4me3. In a first overall approach, the percentage of H3K4me3 or H3K9me3 associated to HsTIRs from 15 to 30 bp was calculated (Fig. 4a). As a control, the same percentage was calculated for random 30 bp sequences. HsTIRs seemed to be preferentially associated to H3K4me3 marks in HeLa-S3 cells, but this was also found for random 30 bp sequences, preventing any conclusion. Few HsTIRs were found globally associated to H3K4me3 and H3K9me3 marks in Hela-S3 cells, 0.65 and 0.06% respectively (Fig. 4a). This was significantly different (by χ2 test) to what is observed for the random 30 bp sequences (1.8 and 0.4%, respectively), indicating that deposition of H3K4me3 or H3K9me3 marks on HsTIRs were somewhat prevented. Similar analyses were performed for marks near the 3952 TIRs of 30 bp (the best conserved ones), according to their location within genes (HsTIR in) or in intergenic regions (HsTIR out), with no significant differences between HsTIRs (whatever their location) and random sequences (Fig. 4b). This suggests that divergent TIRs previously analysed do not behave as the most conserved ones towards histone modifications, the latter being closer to the random sequences.

Another overall approach was used to detect whether HsTIRs (of 15 to 30 bp) are associated or not with H3K4me3 or H3K9me3 marks. Here, both histone modifications were searched within 20-kb windows around TIR sequences by 2 kb intervals (Fig. 4c). Data only showed H3K4me3 marks being not preponderant over H3K9me3 as is known for the whole genome. Similar analyses were performed for the 3952 TIRs of 30 bp according to their locations. No differences were detected between marks and/or according to TIRs locations. Once again, the regulation of divergent (old) and conserved TIRs seems to differ, the latter being more similar to the random 30 bp sequences (or whole genome).

To complete the analyses, the percentage of *HsMar1* full-length copies associated to H3K4me3 or H3K9me3 marks was calculated. Among the 231 copies, only a few were found associated to the expected marks (0.43% for H3K4me3, i.e. 1 copy and 0.96% for H3K9me3, i.e. 2 copies). These results differed highly from randomly chosen control genes (79 and 48% respectively)(Fig. 4d), but also from the recombinant copy, where H3K9me3 are predominant. In addition, the repartition of both marks on each sides of *HsMar1* (Fig. 4e) confirmed that there was no specific mark associated to endogenous *HsMar1*, in a 20 kb window around the copies.

Finally, we examined the H3K4me3 and H3K9me3 marks associated to *SETMAR*. ChIP were done on HeLa-S3 cells and completed by ENCODE ChIP-seq results available on HepG2, MCF7 and PC-3 cell lines (data not shown). All revealed that *SETMAR* promoter was marked by H3K4me3 but not by H3K9me3 (Fig. 4f).

## Discussion

Interactions between host-genomes and TEs have been studied during the two past decades regarding the potential of TEs to create regulatory networks via the coding of various regulatory elements that enables them to regulate genes expression [1, 24]. The most and best characterized in human are the LINEs L1 since many of them are still active and their impact on genome regulation and human health of high interest. TEs have commonly been found as largely repressed in somatic cells, however the recognition/identification mechanisms used by the cell have been unsolved, the most shared hypothesis being the involvement of small RNA encoded by old elements [25]. Conversely, a cutting edge issue concerns the biological role of TEs, with increased evidence for the involvement of some “active” TEs in cell differentiation and early development [24, 26, 27]or their reactivation in cancer cells [28]. The regulation of intra-genomic transposition of TEs remains little studied, especially for DNA transposons [14, 29]. The *HsMar1*/SETMAR family of DNA transposons offers an opportunity to approach these questions in human: first, it is a unique example of network in which the transposon supplies both components, the TIRs being the regulatory elements, and the regulatory protein being potentially SETMAR. Second, the ancestral element being reactivated [19], it is now possible to mimic the early steps of *HsMar1* invasion in a naive genome, but also to highlight regulations that are implemented during *HsMar1* reactivation in a genome which already contains “old” copies. Providing a recombinant *HsMar1* transposase in *trans*, we demonstrate that the excision (the first step of transposition) is prevented in human cells while it works in cells naive for *HsMar1* (CHO in our study). This clearly demonstrates that beyond the transposase expression regulation, the mechanism of the transposition itself is regulated for TEs transposing via a DNA intermediate in somatic cells. Inactivation of de novo *HsMar1* elements in human cells is mediated by CpG methylation and deposition of H3K9me3 marks that are conventional for TEs inactivation [5]. This mechanism prevents the transposition in *trans* of conserved copies and raises two issues: how does the cell “recognize” the recombinant active copy and what is the regulation of the old, degenerated copies? An attempt to answer the first question could be provided by naive CHO cells since they are able to prevent *HsMar1* excision, sustaining a role for piRNA acting in *trans* in human TEs regulation [30, 31]; in Drosophila ovaries, PIWI-piRNA complexes repress TEs by modifying the chromatin state, such as by H3K9me3 [32, 33]. This hypothesis might be widened to somatic cells because they express PIWI-like proteins [34, 35] and because HeLa cells were demonstrated to contain piRNAs [36]. Beyond these specific small RNAs that are well known to regulate TEs activity, further evidence grows up indicating that TEs are source of various RNA (sRNA or lncRNA) that may interfere both with gene expression and TEs control (review in [1]). A non-exclusive possibility involves TRIM28, a master regulator of transposable elements in human, that establish H3K9me3 at TEs loci, jointly with KRAB-ZFPs [37].

To raise the second issue, we chose to analyse the epigenetic status of endogenous *HsMar1*. Overall approaches have indicated that both *HsMar1* promoters and TIRs are hypo-methylated, probably the result of former methylations, followed by CpG shortage undergoing at position 24–25 of TIRs. In addition, endogenous *HsMar1* display specifically low modifications of histone marks when compared to the whole genome (difference of a factor about ten), but respecting a prevalence of H3K4me3 over H3K9me3. Finally, the SETMAR gene display the same epigenetic regulation as others human housekeeping genes. The fact that *HsMar1* has escaped host silencing after the primitive burst of amplification may appear counterintuitive, but many studies indicate that TEs are not as robustly silenced as commonly assumed (for a review see [1]). In addition, it is coherent with the fact that at least some of the old copies are transcribed (Fig. 1c); we also know that many MITEs derived from *HsMar1* are efficiently transcribed, giving siRNA identified as hsa-mir-548 [18]. Nevertheless, we have only performed an overall approach, which could mask local variations in specific context to gene expression profiles (cell cycle, tissue-specificity, pathology context, etc.…). Further studies are needed to verify whether certain loci could be differentially regulated.

In their recent publication, Chuong et al. [1] exemplify the idea that TEs are a prolific source of biochemical regulatory activity in host cells. They review recent findings sustaining the hypothesis that TEs have catalysed the evolution of gene-regulatory networks. Given its significant role in the maintenance of genomic stability, and the presence of about 4000 still efficient binding sites around the human genome, SETMAR constitutes a strong candidate to implement such a network [38]. With *HsMar1*, it constitutes a textbook case of TE-genome interaction: old elements could be both the source of regulation to prevent de novo insertions and (via the TIRs) the *cis*-elements of the regulatory network, and SETMAR (after being domesticated) could be the actor of the regulation.

## Conclusions

Our work highlights that de novo and old *HsMar1* are differently regulated by epigenetic mechanisms. The mechanism that prevents the amplification of de novo insertions is highly efficient, suggesting at least an action in *trans* that may be supported by RNA interference. Old *HsMar1*, that could constitute the SETMAR network, are generally detected as lacking epigenetic marks, whatever their location relative to the genes. Despite the growing number of studies that have demonstrated SETMAR to be a main actor of genome stability in human cells, and the TIRs to be efficient binding sites, there are yet no data confirming that SETMAR may influence nearby gene expression. Future studies need to address this question, assessing this so promising network.

## Methods

### DNA

The whole *HsMar1* excision cassette (Fig. 1a) and the HSMAR-RA ORF ([19]) were synthetized by Eurofins and cloned in pBluescript KS+. The plasmid containing the *HsMar1* cassette is named pHsMar1. The mPB plasmid, expressing PiggyBac transposase, was obtained from the Welcome trust Sanger institute ([39]).

HSMAR-RA ORF was cloned in fusion with Maltose Binding Protein (MBP) in pMalc2 (New England Biolabs). The resulting fusion MBP-HSMAR-RA and HSMAR-RA were cloned in pCS2 plasmid. After PCR amplification, CRE ORF was cloned in pCS2.

All constructs were controlled by sequencing (Eurofins MWG Biotech). DNA preparations to be transfected were performed with Nucleobond Xtra EF kit (Macherey-Nagel).

Genomic DNAs needed for analyses were extracted and purified with Nucleospin Tissue kit (Macherey-Nagel).

PCR primers used in the study are listed in Additional file 1: Table S1 and were purchased by Eurofins.

### Recombinant cell lines containing the *HsMar1* excision cassette

HeLa (human, #ACC 57- DSMZ) and CHO (Chinese hamster ovary, #ACC-110 DSMZ) cells were cultured as recommended by the Leibniz Institute DSMZ German Collection of Microorganisms and Cell Cultures. Transfections were performed at 60–70% cell confluence with JetPEI (Polyplus transfection). To prepare recombinant HeLa and CHO cell lines (containing the excision cassette), cells were transfected with 1 μg of mPB and pHsMar1. 48 h post-transfection, 1/10 of transfected cells were seeded in B10 plates with medium supplemented with 1 μg/ml or 10 μg /ml puromycin for HeLa and CHO cells respectively. After 2 weeks of selection, individual clones were selected and amplified.

### Western blot

Cells were resuspended in Laemmli buffer. Proteins were separated in SDS-PAGE, transfered onto Hybond-ECL membrane (GE Healthcare), probed with MBP antibody (New Englands Biolabs) and revealed with HRP secondary antibody with ECL reagent (GE Healthcare).

### Excision assays

Excision assays were performed after 5 to 7 passages for individual established clones. 100,000 cells of recombinant HeLa or CHO were transfected with JetPEI and 150 or 1050 ng of pCS2-MBP-HSMAR-RA or 500 ng of pCS2-CRE, completed to 2 μg with pCS2 to prevent data variations due to transfected DNA amount variations. Control assays were performed with 2 μg of pCS2 or pCS2-GFP. The expression of GFP depends on its juxtaposition of the CMV promoter next to the GFP ORF which contains the GFP ATG. The in silico reconstitution of HsMar1-GFP fusion transcript did not allow the translation of GFP.

GFP expression (for control or after excision) was observed by photonic microscopy 3–5 days later. The excision sites obtained after transposition or CRE recombination (HeLa or CHO recombinant cell lines) were amplified by PCR with e-primers using 50 ng of genomic DNA and FlexiTaq, according to the manufacturer (Promega). PCR products were cloned in pGEMT and sequenced by Eurofins MWG Biotech.

### Methylation analysis

500 ng of genomic DNA (from recombinant CHO and HeLa cell lines) was treated with sodium bisulfite according to Epimark bisulfite conversion kit (New England Biolabs). Fragments adjacent to the 5′ and the 3′ recombinant *HsMar1* TIRs were amplified using 50 ng of treated DNA and the m-primers designed by Methprimer (Additional file 1: Table S1). PCR products were cloned in pGEMT and sequenced by Eurofins. Analysis of CpG methylations was done using the QUMA software. ENCODE RRBS analysis were used to detect methylation environment (ENCFF001TMU & ENCFF001TMV) [40].

### Detection of histone marks

Chromatin was extracted from recombinant HeLa and CHO cell lines according to Browne’s protocol ([41]). ChIPs were performed with anti-Histone H3K9me3 (abcam ab8898) and anti-Histone H3K4me3 (abcam ab8580) antibodies using a ChIP kit (abcam ab500). qPCR was then performed on 20 ng of ChIP DNA, in order to detect *GAPDH*, *TAF7* and *EIF4a* promoters, 5′ and 3’ *HsMar1* adjacent sequences, using the c-primers (Additional file 1: Table S1) and the Mesa Green qPCR Master SYBR Green I (Eurogentec), in a BioRad Opticon instrument. Quantitative data were recovered using the BioRad CFX Manager software. ChIP assays were performed in triplicates. The *GAPDH* housekeeping gene was used as the endogenous normalizer. RQ was calculated using the conventional method of the ΔΔCt, where RQ (Relative Quantification) = 2-Δ ΔCt.

ENCODE ChIP-seq data were used for histone marks analysis (H3K9me3 and H3K4me3) of HeLa-S3 cells (ENCFF712ATO, ENCFF310XFO) [40]. *HsMar1* TIRs and complete *HsMar1* sequences were retrieved form hg38 assembly using Geneious software. We found 12,378 TIRs of which 3952 were 30 bp long, and 231 complete *HsMar1* (length 1–1.3 kb). Intersect and Fetch Closest Feature tools available on the Galaxy plaform “Galaxeast” were used to analyse the association of full-length or *HsMar1* TIRs with the epigenetic marks H3K4me3 and H3K9me3. *HsMar1* TIR inside genes were defined as containing as least one base in common with transcribed genes, i.e. coding protein, miRNA and lncRNA genes. The transcripted genes were retrieved from gencodebasic-v27 UCSC database. *HsMar1* TIR outside genes corresponded to TIRs excluded from the precedent criteria. To verify whether the association of the different histone marks with *HsMar1* TIRs was random or not, 2000 random 30 bp sequences were extracted from hg38 reference human genome whatever their sequences and locations by an in home-made program written in R (R development Core Team, 2017). The only constraint was that the number of random 30 bp sequences issued from each chromosome is proportional to the length of the chromosomes. These sequences were analysed as done for *HsMar1* TIRs for their association with H3K4me3 or H3K9me3. 250 random genes were extracted with the Galaxy “random select lines” tool from the gencodebasic-v27 UCSC database.

### Statistical tests

The percentage of TIRs/histone marks association between *HsMar1* TIRs or shuffle TIRs were compared by a χ2 test. The distributions of histone marks around TIRs or full-length *HsMar1* copies were compared by a χ2 test.

## Additional file

Additional file 1: **Data S1.** Recombinant *HsMar1* insertion sites in HeLa and CHO cell lines. **Data S2.** Inter-plasmidic active transposition of HSMAR-RA. **Table S1.** Primer list. (DOCX 123 kb)

## Abbreviations

ChIP: Chromatine immuno-precipitation
CHO: Chinese Hamster Ovary
CRE: Causes recombination protein
DNA: Deoxyribonucleotide acid
EIF4: Eucaryotic initiation factor 4
ENCODE: Encyclopedy of DNA elements
ES: Embryonic Stem
GAPDH: Glyceraldehyde 3 phosphate deshydrogenase
GFP: Green fluorescent protein
H3K4me3: Histone 3 lysine 4 trimethylated
H3K9me3: Histone 3 lysine 9 monomethylated
H4K16me: Histone 4 lysine 16 monomethylated
H4K20me3: Histone 3 lysine 20 trimethylated
HRP: Horseradish peroxydase
KRAB-ZFP: Krüppel box associated-zinc finger protein
LINEs: Long Interspersed Sequences
lncRNA: Long non coding RNA
loxP: CRE recombination sites
mCpG: Methylated CG dinucleotide
miRNA: Micro RNA
MITE: Miniature transposable elements
My: Million Years
ORF: Open reading frame
PB: Piggy Bac
pCMV: Cytomegalovirus promoter
PCR: Polymerase chain reaction
piRNA: Piwi associated RNA
PIWI: piRNA interacting protein
qPCR: Quantitative PCR
RNA: Ribonucleotide acid
RNAi: RNA interference
RRBS: Reduced Representation Bisulfite Sequencing
RT-PCR: Reverse transcription-PCR
SB: Sleeping Beauty
SDS-PAGE: Sodium dodecyl sulphate polyacrylamide gel electrophoresis
siRNA: Small interfering RNA
sRNA: Small RNA
TAF7: TATA box associated factor 7
TE: Transposable Element
TIR: Terminal Inverted Repeats
TRIM28: Tripartite motif containing 28
UCSC: University California Santa Cruz
