# Supplementary material for: The epigenetic regulation of HsMar1, a human DNA transposon
Source: BMC Genet. 2019 Feb 14;20:17. doi: 10.1186/s12863-019-0719-y (PMC6375154; doi:10.1186/s12863-019-0719-y)
Supplement: Supplementary file 1 — Data S1. Recombinant HsMar1 insertion sites in HeLa and CHO cell lines. Data S2. Inter-plasmidic active transposition of HSMAR-RA. Table S1. Primer list. (DOCX 123 kb) [file 12863_2019_719_MOESM1_ESM.docx]

**The epigenetic regulation of *HsMar1*, a human DNA transposon**

**Renault *et al -* Supplemental data**

**Supplemental data #1: Recombinant *Hsmar1* insertion sites in HeLa cells.**

Genomic DNA of 3 HeLa and 3 CHO recombinant cell lines were extracted and purified with Nucleospin Tissue kit (Macherey-Nagel). The number of inserted cassette per cell was estimated by qPCR relative to the number of GAPDH gene, according that there was one copy of GAPDH gene per haploid genome in HeLa and CHO cells (“q-primers” in Table #S1). Amplifications were done using the Mesa Green qPCR Master SYBR Green I, following the instructions of the manufacturer (Eurogentec) on a BioRad Opticon instrument. Quantitative data were recovered using the BioRad CFX Manager software. Genomic DNAs were assayed in triplicates. The results show that CHO cell lines have only one integrated HsMar1 cassette and HeLa cell lines, one or two integrations (see below).

The location of insertions was determined for 3 CHO and 3 HeLa cell lines by I-PCR (“i-primers” in Table #S1) using the ThermoTaq according to the manufacturer (Eurobio), followed by cloning in pGEMT and sequencing. Insertion sites sequences were checked for the presence of Piggybac TIRs at both ends and aligned on human and Chinese hamster genomes using BlastN software (NCBI). The presence of PiggyBac TIRs was detected in each CHO and HeLa cell lines confirming the insertion of the HsMar1 cassette in genome. The sites of integration of the cassette are presented for HeLa cell lines in the following table. No significant homology of integration sites of CHO recombinant cell lines were obtained with the available *Cricetulus griseus* (Chinese hamster) draft genome due to the weakness of the genomic sequences available in this species.

| Line references | Number of insertion | Genomic location |
| --- | --- | --- |
| HeLa-B4 | 2 | Chr 17 – 88617  Chr 6 - 218306 |
| HeLa-D2 | 1 | Chr 10 - 289555789 |
| HeLa-D4 | 1 | Chr 5 - 70021803 |

**Supplemental data #2: HSMAR-RA is active in HeLa cells 48H post-transfection.**

To verify if the lack of *HsMar1* excision associated to HeLa cells in the main text could be correlated to a lack of HSMAR-RA expression or activity in these cells, two kinds of assays were set-up. First, the recombinase expression was simply verified by western blotting, using an antibody directed against the MBP-tag flanking the expressed transposase. This approach was made necessary for 2 reasons: (1) no anti-HSMAR-RA antibodies are available and (2) *mariner* transposases require such a tag for increased activity and stability without altering their properties (Dussassois et al, 2017). Second, the recombinase activity in HeLa cells was verified by an plasmid-to-chromosome transposition assay.

**A.** HSMAR-RA expression in recombinant HeLa cell line (Hela-D2) after transfection with 0, 150 and 1050 ng of expressing plasmid (pCS2-MBP-HSMAR-RA).

| **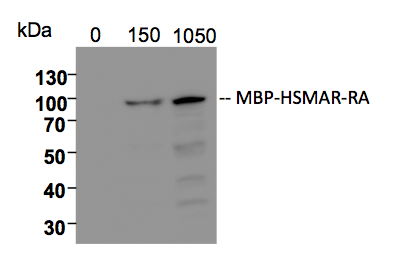** | 48H post-transfection, cells are collected, disrupted in Laemmli solution and the resulting crude extracts are run onto an SDS-page. After transfer, an anti-MBP antibody (New England Biolabs) reveals the recombinant protein. The amount of the expected recombinant protein (MBP-HSMAR-RA, of about 85 kDa) relies on the amount of transfected DNA. |
| --- | --- |

**B.** Plasmid-to-chromosome transposition assays are performed using two different HSMAR-RA constructs, expressing either MBP-HSMAR-RA (pCS2l-MBP-HSMAR-RA used in A) or HSMAR-RA (pCS2-HSMAR-RA) and a transposition cassette containing the promoter of SV40- neomycine resistance gene surrounded with the 5’ITR-UTR and 3’ITR-UTR of Hsmar1.

Plasmids expressing or not the recombinase are co-transfected in various amount (0, 500 or 1000 ng as indicated) with the transposition cassette donor DNA (500ng), in HeLa or CHO cells. 48H post-transfection, recombinant cells are selected against puromycin resistance (see main text, the selection method is the same that used for preparing the excision cassette recombinant cells). 15-days post selection, resistant clones are coloured and counted. The number of resistant clones estimates the transposition efficiency.


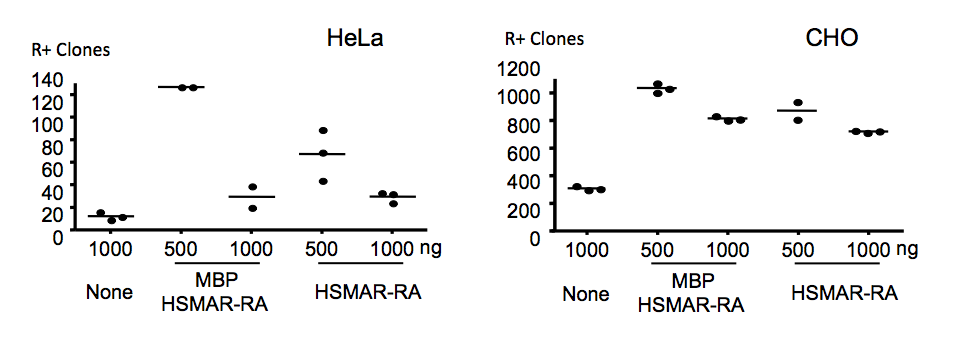


Transposition efficiency is higher in CHO cells (about one order of magnitude) than in Hela cells, and CHO are less sensitive to the amount of produced recombinase as well as to the presence of the MBP-tag. However, these assays demonstrate that HSMAR-RA is active in Hela cells.

Optimal conditions for *HsMar1* transposition are defined using the more sensitive Hela cells. They require the MBP-HSMAR-RA recombinase expressed from 500 ng of transfected expression plasmid. These conditions are retained for the excision assays (main text) after adapting the DNA quantity to cell quantity (*i.e.* 500 ng for transposition correspond to 150 ng for excision).

**Supplemental table #1: Primers used for the study.**

x-primers for RT-PCR, q-primers for qPCR quantification of integrated recombinant *HsMar1*, i-primers for integration site amplification, e-primers for excision fragment amplification, m-primers for methylation analysis after bisulfite conversion, c-primers for ChIP analysis

|  |  | 5'-3' | Tm (°C) |
| --- | --- | --- | --- |
| x primers | Hs ATG | atggaaatgatgttagacaaaaag | 53 |
|  | Hs stop | tcaaaataggaaccattacaatca | 53 |
| q primers | GAPDH Hs up | CCCTTCATACCCTCACGTATTTC | 60 |
|  | GAPDH Hs dw | ATGACAAGCTTCCCGTTCTC | 60 |
|  | GAPDH Cg up | GGACATCAAGAAGGTGGTGAA | 60 |
|  | GAPDH Cg dw | GAGTGGGAGTCACTGTTGAAG | 60 |
|  | GFP up | TCCACACAATCTGCCCTTC | 60 |
|  | GFP dw | GGTGGTCTAGCTTTGTATAG | 60 |
| e primers | GFP-K7 | gcatcaccttcaccctctccactg | 56 |
|  | CMV-K7 | TGGGCGGTAGGCGTGTACGGT | 56 |
| i primers | 5’ITRPB-1 | AATGTCGTAACAACTCCGCC | 54 |
|  | 5’SPLNK-PB#1 | ACCGCATTGACAAGCACG | 54 |
|  | 5’ITRPB-2 | AACTAGAGAACCCACTGCTTAC | 54 |
|  | 5’SPLNK-PB#2 | CTCCAAGCGGCGACTGAG | 54 |
|  | 3’ITRPB-1 | TTCGCTAGCTCGACATGATAAG | 54 |
|  | 3’SPLNK-PB#1 | GTTTGTTGAATTTATTATTAGTATGTAAG | 54 |
|  | 3’ITRPB-2 | TTCTTGCAGCTCGGTGAC | 54 |
|  | 3’SPLNK-PB#2 | CGATAAAACACATGCGTC | 54 |
| m primers | GFP-B5up | TGTTGAAGTTAAGTTTGAGGGAGATAT | 62 |
|  | GFP-B5dw | TTATCTAATAAAAAAACAAAACCATC | 62 |
|  | CMV-A5up | GGGATTTTTAAGTTTTTATTTTATTGA | 62 |
|  | CMV-A5dw | AAACTCTACTTATATAAACCTCCCACC | 62 |
| c primers | EIF4a Hs up | ggcacctcgattagttctcg | 60 |
|  | EIF4a Hs dw | gggaaactccatcgcataaa | 60 |
|  | TAF7 Hs up1 | GTAGACCTGCCCTGTGTTATG | 60 |
|  | TAF7 Hs dw1 | CTCCACAGGAGGATAGAGATCA | 60 |
|  | GAPDH Hs up | CCCTTCATACCCTCACGTATTTC | 60 |
|  | GAPDH Hs dw | ATGACAAGCTTCCCGTTCTC | 60 |
|  | EIF4a Cg up | GATCTCCAGGAACGCTGTAATA | 60 |
|  | EIF4a Cg dw | ATGTGCTCCTCGGATTTGT | 60 |
|  | TAF7 Cg up | GGGAGATAATTGCTGAGGATGAA | 60 |
|  | TAF7 Cg dw | CATCATGTTCTAGGGAGTCGTG | 60 |
|  | GAPDH Cg up | GGACATCAAGAAGGTGGTGAA | 60 |
|  | GAPDH Cg dw | GAGTGGGAGTCACTGTTGAAG | 60 |
|  | CMV up | CAAGTACGCCCCCTATTGAC | 60 |
|  | CMV dw | TATCCACCAGCCCATTGATGTA | 60 |
|  | GFP up | TCCACACAATCTGCCCTTC | 60 |
|  | GFP dw | GGTGGTCTAGCTTTGTATAG | 60 |
